# Comparison of Bioinformatics Pipelines and Operating Systems for the Analyses of 16S rRNA Gene Amplicon Sequences in Human Fecal Samples

**DOI:** 10.3389/fmicb.2020.01262

**Published:** 2020-06-17

**Authors:** Moira Marizzoni, Thomas Gurry, Stefania Provasi, Gilbert Greub, Nicola Lopizzo, Federica Ribaldi, Cristina Festari, Monica Mazzelli, Elisa Mombelli, Marco Salvatore, Peppino Mirabelli, Monica Franzese, Andrea Soricelli, Giovanni B. Frisoni, Annamaria Cattaneo

**Affiliations:** ^1^Laboratory of Neuroimaging and Alzheimer’s Epidemiology, IRCCS Istituto Centro San Giovanni di Dio Fatebenefratelli, Brescia, Italy; ^2^Pharmaceutical Biochemistry Group, School of Pharmaceutical Sciences, University of Geneva, Geneva, Switzerland; ^3^Laboratory of Biological Psychiatry, IRCCS Istituto Centro San Giovanni di Dio Fatebenefratelli, Brescia, Italy; ^4^Institut de Microbiologie de l’Université de Lausanne, Lausanne, Switzerland; ^5^Department of Molecular and Translational Medicine, University of Brescia, Brescia, Italy; ^6^Memory Clinic and LANVIE – Laboratory of Neuroimaging of Aging, University Hospitals and University of Geneva, Geneva, Switzerland; ^7^IRCCS SDN, Naples, Italy

**Keywords:** 16S rRNA amplicon sequencing, QIIME2, bioconductor, UPARSE, mothur, microbiome, fecal human samples

## Abstract

Amplicon high-throughput sequencing of 16S ribosomal RNA (rRNA) gene is currently the most widely used technique to investigate complex gut microbial communities. Microbial identification might be influenced by several factors, including the choice of bioinformatic pipelines, making comparisons across studies difficult. Here, we compared four commonly used pipelines (QIIME2, Bioconductor, UPARSE and mothur) run on two operating systems (OS) (Linux and Mac), to evaluate the impact of bioinformatic pipeline and OS on the taxonomic classification of 40 human stool samples. We applied the SILVA 132 reference database for all the pipelines. We compared phyla and genera identification and relative abundances across the four pipelines using the Friedman rank sum test. QIIME2 and Bioconductor provided identical outputs on Linux and Mac OS, while UPARSE and mothur reported only minimal differences between OS. Taxa assignments were consistent at both phylum and genus level across all the pipelines. However, a difference in terms of relative abundance was identified for all phyla (*p* < 0.013) and for the majority of the most abundant genera (*p* < 0.028), such as *Bacteroides* (QIIME2: 24.5%, Bioconductor: 24.6%, UPARSE-linux: 23.6%, UPARSE-mac: 20.6%, mothur-linux: 22.2%, mothur-mac: 21.6%, *p* < 0.001). The use of different bioinformatic pipelines affects the estimation of the relative abundance of gut microbial community, indicating that studies using different pipelines cannot be directly compared. A harmonization procedure is needed to move the field forward.

## Introduction

The human gut microbiota (GMB) represents the next frontier in medicine to unravel the pathophysiology of chronic diseases and to develop novel treatments. A growing number of studies have shown that human microbial communities can contribute to the development of several pathologies, including cancer (Contreras et al., 2016; Ren et al., 2019), metabolic (Xiao and Zhao, 2014; Cai et al., 2015) and inflammatory (Tedjo et al., 2016; Zhou et al., 2018) disorders, but also brain diseases (Scheperjans et al., 2015; Cattaneo et al., 2017; Horne and Foster, 2018; Rodrigues-Amorim et al., 2018; Winter et al., 2018).

High-throughput sequencing (HTS) technologies have opened new frontiers in microbial community analysis by providing a cost-effective means to identify the microbial phylotypes present in samples (Caporaso et al., 2011). This is commonly achieved by sequencing the 16S ribosomal RNA (rRNA) gene for bacteria and archaea, which contains also specific variable regions that can be used for taxonomic classification. 16S rRNA sequencing allows researchers to get information about the microbial diversity within the gut, to assist in identifying disease-associated microbiome changes. However, the processing of 16S rRNA gene amplicon sequences requires specialized technical and bioinformatic expertise not always accessible to biologists in their own lab. Indeed, the size and complexity of these data, incompleteness of nucleotide databases, and the numerous tools available for each step of the analytical process, make these data difficult to process by untrained users. The analytical post-processing steps required to extract taxonomic information from raw sequences are essentially threefold: (i) merging of read pairs into longer single reads, (ii) quality control and read trimming, and (iii) taxonomic assignment. Each step may need several tools or algorithms, and each of them may require expertise for parameter definition and/or programming and thus may need extensive computational resources and expertise. Furthermore, these bioinformatic tools are not always well-documented, and are often patched together by users, raising doubts on the reproducibility of results (Nekrutenko and Taylor, 2012).

Several workflows have been recently developed to overcome these limitations by simplifying the analytical procedure and allowing untrained users to familiarize themselves with advanced programming or computational techniques. Among the bioinformatic pipelines available so far, those used in QIIME2 (Bolyen et al., 2019), Bioconductor (Callahan et al., 2016b), USEARCH (Edgar, 2010) and mothur (Schloss et al., 2009) are the most widely used to analyze 16S rRNA gene sequencing data. Bioconductor and several plugins in QIIME2 [i.e., DADA2 (Callahan et al., 2016a) and Deblur (Amir et al., 2017)] allow researchers to infer amplicon sequence variants (ASVs) while UPARSE and mothur return operational taxonomic units (OTUs). For ASVs, sequences are resolved down to the level of single-nucleotide differences over the sequenced gene region, while for OTUs sequences that have typically less than 3% of variance from each other were binned into the same OTU.

Although several studies have evaluated the impact of different bioinformatic pipelines on microbial taxonomy, comparative data on real dataset are still missing. Indeed, previous studies analyzed synthetic simulated datasets (Almeida et al., 2018), mock communities (Segota and Long, 2019), chicken (Allali et al., 2017), or rumen (López-García et al., 2018) microbiota. To the best of our knowledge, only three studies analyzed human 16S rRNA gene amplicon sequencing data, but have used the old release of QIIME (D’Argenio et al., 2014; Plummer and Twin, 2016) or did not include Bioconductor in the comparison (Prodan et al., 2020). Finally, as reported for other complex bioinformatic data processing (i.e., neuroimaging analysis), the same pipelines are known to generate different results depending on the computing platform where they are compiled and executed (Gronenschild et al., 2012; Glatard et al., 2015). This variable has never been described in the literature in the context of the analysis of 16S rRNA gene sequencing data.

Based on this, in this study we aimed at evaluating whether different bioinformatic pipelines, and also different OS, can influence the taxonomic classification of the fecal microbiota in 40 human samples. In particular, we analyzed the human 16S rRNA gene sequencing data by using four different pipelines (QIIME2, Bioconductor, UPARSE, and mothur), we ran them using two different OS (Linux and Mac OS), and we compared the results focusing on phylum and genus identification and relative abundance.

## Materials and Methods

### Stool Collection

Stools were collected from participants to an existing larger cohort study on brain aging in 18 memory clinics in Eastern Lombardy, Italy. The parent study aimed at assessing the added value of amyloid imaging in the clinical work-up of patients with cognitive complaints (the Incremental Diagnostic Value of Florbetapir Amyloid Imaging [INDIA-FBP] study) (Altomare et al., 2018). After completion of the INDIA-FBP procedures, 150 patients and controls were asked to further contribute with their samples of stools and blood. As previously reported (Cattaneo et al., 2017), stool samples were collected from subjects at their own home in a sterile plastic cup, stored at −20°C, and delivered to IRCCS Fatebenefratelli Institute in Brescia within the following 24 h, where they have been stored at −20°C until their processing. The stool samples used in the current study were from a subgroup of 40 subjects with cognitive performance from normal to dementia.

The study was approved by the Ethics Committee of “Comitato Etico dell’IRCCS San Giovanni di Dio – Fatebenefratelli” (Brescia, Italy) under registration number 57/2014. Written informed consent was obtained from all participants.

### DNA Extraction

DNA was extracted from 180 to 200 mg of frozen stool using the QIAamp DNA Stool Mini Kit (Qiagen Retsch GmbH, Hanover, Germany) and according to the manufacturer’s instructions. Bead-beating homogenization by TissueLyser II (Qiagen Retsch GmbH, Hanover, Germany) was performed to mechanically disrupt fecal samples before DNA extraction. The samples were homogenized for 10 min at 30 Hz. DNA was then quantified using a NanoDrop ND-1000 spectrophotometer, and then stored at + 4°C for subsequent analyses. All the stool samples were processed at the same time.

### PCR Amplification, Barcoding, and DNA Sequencing

Bacterial DNA was amplified and purified according to 16S Metagenomic Sequencing Library Preparation protocol by Illumina. The first step consists in the amplification of the regions V3 and V4 of the bacterial ribosomal RNA 16S gene, by using the suggested primers (Forward Primer = 5′TCGTCGGCAGCGTCAGATGTGTATAAGAGACA GCCTACGGGNGGCWGCAG; Reverse Primer = 5′GTCTCGT GGGCTCGGAGATGTGTATAAGAGAC AGGACTACHVGGG TATCTAATCC) and the suggested cycling conditions (3′ at 95°C; 25 cycles: 30′′ at 95°C, 30′′ at 55°C, 30′′ at 72°C; 5′ at 72°C). The amplicon DNA was immediately purified with a magnetic bead step, washed in 80% ethanol and resuspended in Tris-HCl 10 mM. Amplicon DNA integrity was assessed by 2.0% agarose gel electrophoresis on gels containing 0.8 mg/mL ethidium bromide and stored at −20°C. Within 1 week from storage, amplicon DNA was uniquely dual-indexed, by using the suggested indices (Nextera XT) and the suggested cycling conditions (3′ at 95°C; 8 cycles: 30′′ at 95°C, 30′′ at 55°C, 30′′ at 72°C; 5′ at 72°C). The resulting indexed DNA was immediately purified with a magnetic bead step, washed in 80% ethanol, resuspended in Tris-HCl 10 mM and stored at −20°C. Within 1 week from storage, indexed DNA was quantified using fluorometric quantification (Qubit, Invitrogen) and the amplicon length was determined by using a Bioanalyzer DNA 1000 chip (Agilent). DNA was then normalized to 4 nM, pooled, denatured with NaOH 0.1N, diluted to the final concentration of 10 pM and loaded into the MiSeq v3 cartridge (Illumina). A paired-end read of 300 cycles per read was performed. Samples from cognitively intact persons and demented patients were sequenced in two different runs. Data are publicly available at www.ebi.ac.uk/ena/data/view/PRJEB35434.

### Bioinformatic Analyses

**FIGURE 1:**
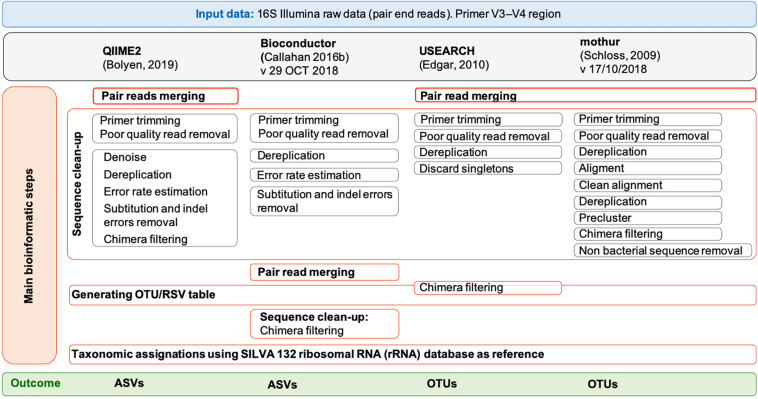
Overview of the pipelines used by free and open-source workflows: QIIME2, Bioconductor, UPARSE, and mothur. Each gray box represents a command of the pipelines. For UPARSE, chimera filtering is part of the OTU clustering step, and OTU taxonomic assignment was performed using mothur.

The paired-end MiSeq Illumina reads (2 × 300 bp) were processed by using four popular tools: QIIME2 (Bolyen et al., 2019) (version 2018.8), Bioconductor (Callahan et al., 2016b) (version 29 October 2018), USEARCH (Edgar, 2010) (version 11.0.667) and mothur (Schloss et al., 2009) (version 1.43.0). For QIIME2, Bioconductor and mothur, the 64 bit version was used while for USEARCH (Edgar, 2010), only a memory-confined 32-bit version is freely available for academic use. QIIME 2 was installed in a conda environment. All the pipelines were run on both a Linux workstation (Ubuntu 14.04.5 LTS) equipped with Intel CPU 8 × 3.70 GHz processors and 31.3 GB of RAM and a MacBook Pro (15-inch, 2018) with 2.6 GHz 6-core Intel Core i7 processor, 16 GB of RAM and macOS Mojave, version 10.14.6. In QIIME2 we followed the “Moving Pictures” tutorial (accessed 3 January 2019), in Bioconductor the pipeline reported in https://f1000research.com/articles/5-1492/v2 (version: 29 October 2018), in USEARCH (Edgar, 2010) the instruction of the online tutorial^1^ (accessed 4 January 2019), and in mothur the SOPs in https://www.mothur.org/wiki/MiSeq_SOP (accessed 09/27/2018). Details for each pipeline are shown in Figure 1 and the list of commands used for each tool is reported in Supplementary Methods 1–6. Briefly, sequencing Illumina MiSeq data were already demultiplexed. The parameters used for the quality check of the 16S rRNA data were those suggested in the corresponding tutorials. In general, forward and reverse primers, reads containing ambiguous bases or homopolymers greater than eight base pairs in length as well as chimeras were removed in all pipelines. Moreover, in QIIME2 we set a maximum number of expected errors equal to 2 and reads truncation if the quality score was less than 2. In Bioconductor we used a maximum of 2 and 5 expected errors per-read for forward and reverse reads, respectively, and reads truncation if the quality score was less than 2; moreover, features with ambiguous phylum annotation were discarded. For both QIIME2 and Bioconductor, the software packages Divisive Amplicon Denoising Algorithm 2 (DADA2) (Callahan et al., 2016a) was used to infer true biological sequences from reads. Since the samples were sequenced in two different runs and that different runs may have different error profiles, the denoising process was applied separately to the two runs. In QIIME2, the “dada2” plugin uses the DADA2 R library and has been implemented to perform sequence denoise, dereplication, and chimeras filtering. All these steps correspond to individual commands in Bioconductor. For QIIME2, the pipeline included also the extraction of the representative sequences using “feature-table” and their classification by taxon using the “feature-classifier” (Supplementary Methods 1). For Bioconductor, the pipeline included also the taxonomic classification of the ASVs using “assignTaxonomy,” the construction of the phylogenetic tree using the *phangorn* R package (v2.4.0) (Schliep, 2011) and the creation of a single data object that can be used for graphical and statistical purposes using *phyloseq* R package (v1.24.2) (McMurdie and Holmes, 2013) (Supplementary Methods 2). The UPARSE (Edgar, 2013) and the UNOISE (Edgar, 2016) pipelines are both implemented in USEARCH and have been used to defined OTUs with a similarity threshold of 97 and 99%, respectively, as recommended by the author of USEARCH^2^. All the steps preceding the clustering, namely merging, filtering (where a maximum number of expected errors equal to 1 was set) and dereplication, are the same between the two pipelines (Supplementary Methods 3). In mothur, sequences were merged using “make.contig” command, quality filtered by “screen.seqs” command, dereplicated, aligned to the reference database SILVA v132 (Quast et al., 2013) and pre-clustered allowing 1 difference for every 100 bp of sequence (four differences in our case) (Supplementary Methods 4). After removing the undesirables features (i.e., chloroplasts, mitochondria, archaea, eukaryota, or unknown) using the “remove.lineage” command and chimera by applying the “chimera.vsearch” command, unique sequences were clustered into OTUs at 97 and 99% identity.

SILVA (version 132) (Quast et al., 2013)^3^ and RDP (version 16) reference databases were customized following the instructions on the respective tutorials for QIIME2 and mothur (Supplementary Methods 5, 6) and were downloaded for Bioconductor (SILVA v132^4^, RDP v16^5^). As SILVA version 132 was not available for UPARSE, taxonomic assignment of OTUs was performed on mothur (Supplementary Method 3), where bacterial classification identity was set to 80%. ASVs and OTUs found in a single sample (also named as singletons) were discarded.

In order to facilitate the data analysis and reproducibility, the list of all commands used for each pipeline and the number of reads assigned to taxa, are included as Supplementary Methods 1–6 and Supplementary Table 1.

### Statistical Analysis

All statistical analyses and graphs were performed using GraphPad Prism Software (v 8.1.1) (GraphPad Software, San Diego, CA, United States) except for Venn diagrams that were created with the jvenn web application^6^ (Bardou et al., 2014). Differences among the four used pipelines, in term of the number of reads assigned, the number of identified phylum and genera as well as their relative abundances were tested using Friedman rank sum test conducted with a Dunn’s correction. Wilcoxon signed rank test was applied when genera relative abundances were compared between two pipelines. Significance level of alpha = 0.05 was used.

## Results

### Impact of the Use of Different Pipelines on Data Output

A total of 4715000 reads from 40 fecal samples were used to infer and to compare the taxonomical composition identified by the pipeline suggested in QIIME2, Bioconductor, UPARSE/UNOISE and mothur. The processing of the samples took approximately 3 h of computational time in QIIME2, approximately 8 h in Bioconductor, less than 1 h in UPARSE and approximately 9 h in mothur (Table 1). The comparison of the pipelines between Linux and Mac OS showed that QIIME2 and Bioconductor provided identical outcomes, negligible differences in the number of reads assigned at any identity were obtained when using UPARSE or mothur (*p* > 0.999).

**TABLE 1:** Results of 16S sequencing analyses by using QIIME2, Bioconductor, UPARSE, or mothur.

	**QIIME2**		**Bioconductor**		**UPARSE**		**Mothur**		***p-Value^c^***
	**Linux**	**Mac**	**Linux**	**Mac**	**Linux**	**Mac**	**Linux**	**Mac**	
Software version	2018.08		R version 3.5.1 dada2 v 1.8.0 phyloseq v 1.24.2 phangorn v 2.4.0		v11.0.667_i86 linux32	v11.0.667_i86 osx32	v.1.43.0	v.1.43.0	
Approximate analysis time	4 h	3 h	9 h	8 h	45 min	45 min	9 h	9 h	–
# input reads	4715000								
# reads after filtering/denoising	3,391,670		3,736,927		3,173,733		3,244,489		–
# reads assigned at phylum level^a^ (tot n, mean ± SD)	2,941,772 (87%) 73,544 ± 40,280		3,143,413 (84%) 78,585 ± 37,730		3,123,028 (98%) 78,076 ± 46,872	3,131,211 (99%) 78,280 ± 46,973	2,812,333 (89%) 70,308 ± 35,353	2,812,470 (87%) 70,312 ± 35,356	<0.001
# unclassified reads at phylum level (tot n, mean ± SD)	3,567 (< 1%) 89 ± 210		163 (< 1%) 4 ± 7		77,395 (2%) 1,935 ± 5,119	79,031 (2%) 1,976 ± 5,130	47,883 (2%) 1,161 ± 4,046	48,770 (2%) 1,219 ± 4,048	<0.001
# reads assigned at genus level^b^ (tot n, mean ± SD)	2,770,029 (82%) 69,251 ± 37,324		2,798,953 (75%) 69,974 ± 32,881		3,123,028 (98%) 78,076 ± 46,283	3,131,211 (99%) 78,280 ± 46,382	2,812,333 (89%) 70,310 ± 34,911	2,812,470 (87%) 75,717 ± 12,956	<0.001
# unclassified reads at genus level (tot n, mean ± SD)	171,743 ^b^ (5%) 4,294 ± 4,139		Taxonomic filtering during processing		801,052 (25%) 20,026 ± 16,033	923,329 (29%) 23,083 ± 19,208	763,315 (24%) 19,083 ± 13,009	751,717 (23%) 18,793 ± 12,956	<0.001

*QIIME2 and Bioconductor gave identical outcomes on both operating systems. Mothur failed to complete the pipeline on Mac. Percentage are calculated using the # of reads after filtering/denoising as denominator. ^*a*^Reads assigned any bacteria identity (including “unclassified”) but excluding Eukaryota and Archaea. ^*b*^For QIIME2 we considered as unclassified sequences those with incomplete taxonomies (terminating at any level with ‘__’). ^*c*^p-Values using Friedman test followed by Dunn’s multiple comparisons test.*

Overall, after filtering and denoising, pipelines in QIIME2 and Bioconductor resulted in a higher number of reads to assign (QIIME2: 3391670; Bioconductor: 3736927; UPARSE: 3173733, regardless the OS; mothur: 3244489, regardless the OS). Among these reads, 84–99% were assigned at the phylum level and 75–99% at the genus level, depending on the pipeline used. Similarly, the proportion of reads assigned to the Bacteria domain, but with no taxonomical assignment at phylum and genus level, differed dramatically among pipelines. Excluding Bioconductor, where the removal of unclassified reads is one step included in the pipeline, the proportion of unclassified reads at the genus level varied from 5% in QIIME2 to 24–29% in UPARSE and mothur (*p* < 0.001, df = 4, X^2^ = 103). UPARSE-linux provided the same number of unclassified reads at the phylum level as those obtained by using UPARSE-mac (*p* > 0.999), and the same number of unclassified reads at the genus level as those obtained with mothur, regardless the OS (*p* > 0.999).

### Impact of the Use of Different Pipelines on Phylum Taxonomic Identification

**FIGURE 2:**
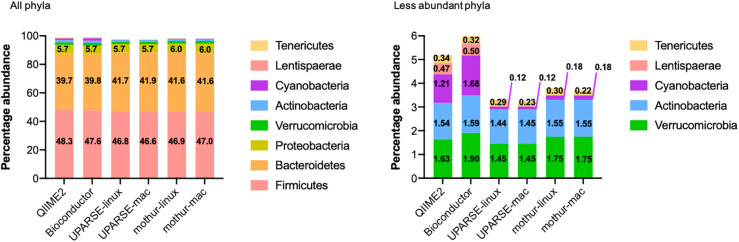
Phyla distribution as identified by using QIIME2, Bioconductor, UPARSE, or mothur. The phyla present in less than 0.005% were *Epsilonbacteraeota* (QIIME2: 0.0046%, Bioconductor: 0.0043%, UPARSE-linux: 0.0049%, UPARSE-mac: 0.0049%, mothur: 0.0050%) and, for mothur only, *Planctomycetes* (0.0006%), *Acidobacteria* (0.0003%), *Nitrospirae* and *Gemmatimonadetes* (0.0001% both) *Chloroflexi* and *Omnitrophicaeota* (<0.0001%, both).

**FIGURE 3:**
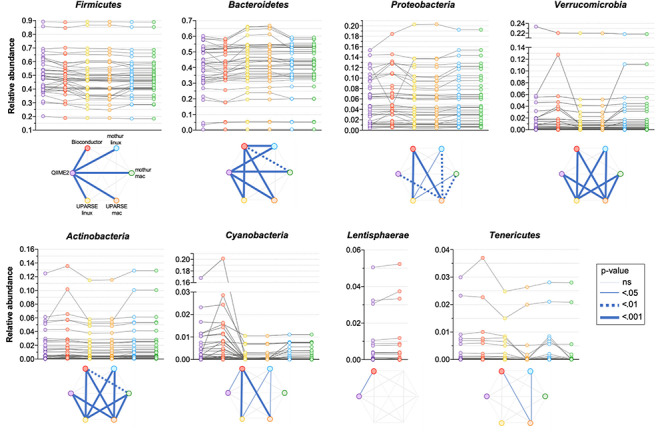
Comparison of the relative abundance of phyla obtained by using QIIME2, Bioconductor, UPARSE, or mothur. *p*-Values were calculated using Friedman test followed by Dunn’s multiple comparisons test. Wilcoxon signed rank test was applied when only two pipelines were compared.

All the used pipelines showed general agreement in the identification of the phyla distribution: the proportion of *Firmicutes* varied from 46.6 (UPARSE-mac) to 48.3% (QIIME2), *Bacteroidetes* from 39.7 (QIIME2) to 41.9% (UPARSE-mac), *Proteobacteria* from 5.7 (QIIME2, Bioconductor, UPARSE) to 6.0% (mothur), *Verrucomicrobia* from 1.45 (UPARSE, regardless the OS) to 1.90% (Bioconductor), *Actinobacteria* from 1.44 (UPARSE-linux) to 1.59% (Bioconductor) and, *Tenericutes* from 0.22 (mothur-mac) to 0.34% (QIIME2) (Figure 2). *Cyanobacteria* were detected in lower abundance by UPARSE (0.12%, regardless the OS) and mothur (0.18%, regardless the OS) as compared to Bioconductor (1.68%) and QIIME2 (1.21%), whereas *Lentisphaerae* were identified only by using QIIME2 and Bioconductor (0.47 and 0.50%, respectively). Pairwise comparisons among pipelines revealed widespread differences in the relative abundance of all the phyla (*p* < 0.0002) (Figure 3). UPARSE (on Linux and Mac OS) or mothur (on Linux and Mac OS) provided comparable values (*p* > 0.999). Similar results were obtained clustering OTUs at 99% (Supplementary Figure 1).

### Impact of the Use of Different Pipelines on Genus Taxonomic Identification

**FIGURE 4:**
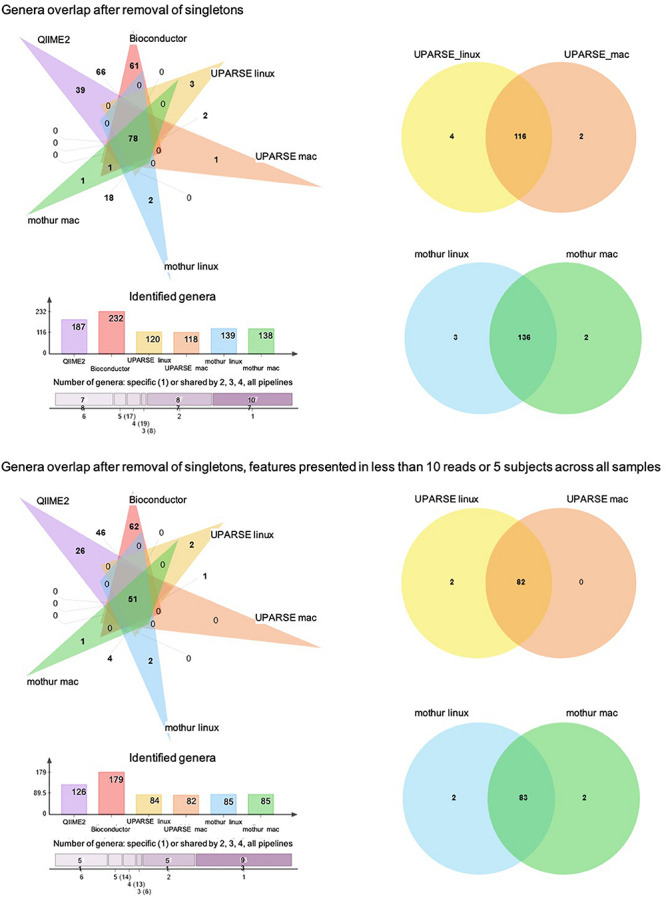
Venn diagram showing the number of shared and specific bacterial genera among the pipelines. Histogram representing the number of genera identified by each pipeline and number of genera shared between 6, 5, 4, 3, 2, and no pipelines are also reported.

At the genus taxonomic level, QIIME2 and Bioconductor identified a higher number of bacterial genera than those obtained by UPARSE-linux, UPARSE-mac, mothur-linux, and mothur-mac (*p* < 0.001, df = 5, X^2^ = 125) (Figure 4A). After singleton removal, 187 genera were identified by QIIME2, 232 by Bioconductor, 120 by UPARSE-linux, 118 by UPARSE-mac, 139 by mothur-linux, and 138 by mothur-mac. Considering all the 316 genera identified, 78 were in common to all the different pipelines, 17 were in common to 5, 19 were in common to 4, 8 were in common to 3, and 87 were in common to 2 out of 6 pipelines. In terms of absolute number of shared genera, QIIME2 and Bioconductor were the two pipelines that provided the most similar results, sharing 147 genera, while UPARSE-mac and QIIME2 were the most different ones, sharing 78 genera. In terms of proportion of shared genera, UPARSE-linux and UPARSE-mac as well as mothur-linux and mothur-mac, were the most producing the overlapping results. Similar results were found after removal of the genera identified in less than five subjects or with less than 10 reads across all samples (Figure 4B).

**FIGURE 5:**
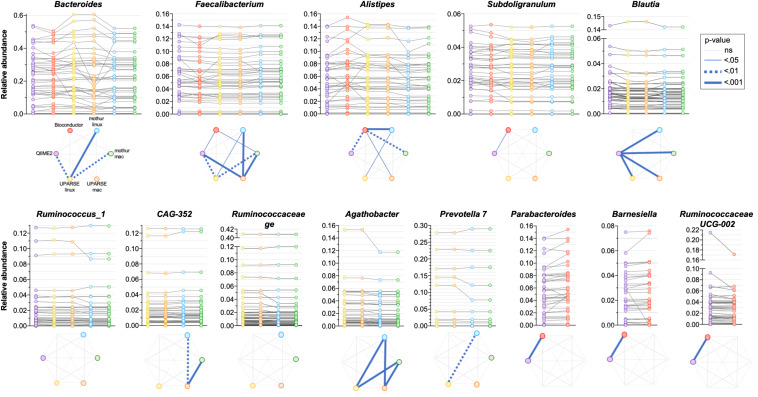
Comparison of the relative abundance of the 10 most abundant genera obtained by applying QIIME2, Bioconductor, UPARSE, or mothur at genera level. *p-*Values were calculated using Friedman test followed by Dunn’s multiple comparisons test. Wilcoxon signed rank test was applied when only two pipelines were compared.

As the quantitative comparison of all the genera was unfeasible, we arbitrarily selected and compared the 10 most abundant genera identified in each pipeline. All the used pipelines showed general agreement in the identification of the most abundant genera. It has been found that *Bacteroides*, *Faecalibacterium*, *Alistipes* and *Subdoligranulum* were consistently identified by all the pipelines (Figure 5). Moreover, *Blautia* and *Ruminococcus_1* were concordantly identified by QIIME2, mothur-linux, mothur-mac, UPARSE-linux and UPARSE-mac; *CAG-352*, *Ruminococcaceae ge*, *Agathobacter* and *Prevotella 7* were concordantly identified by mothur-linux, mothur-mac, UPARSE-linux and UPARSE-mac and, *Parabacteroides*, *Barnesiella* and *Ruminococcaceae UCG-002* were concordantly identified by QIIME2 and Bioconductor. *Eubacterium coprostanoligenes group* was identified by QIIME2 only while *Lachnosphira*, *Ruminococcus 2*, and *Lachnospiraceae NK4A126 group* by Bioconductor only. Pairwise comparisons among pipelines revealed widespread differences in the relative abundance of above-mentioned shared genera. Indeed, pipeline-dependent differences in term of relative abundance was reported for all (*p* < 0.041) except for *Ruminococcus_1* and *Ruminococcaceae* (*p* > 0.074). Among the same 10 genera identified as most abundant by UPARSE-linux and UPARSE-mac, a trend forward significance was reported for *CAG-352* only (*p* = 0.056). Similar results were obtained after clustering OTUs at 99% (Supplementary Figure 2).

The 10 genera identified as the most abundant from the different pipelines were selected also when RDP was used as a reference database. Six out of 10 genera were consistently found in all pipelines and the 3 most abundant genera, *Bacteroides, Faecalibacterium* and *Alistipes*, have also been identified using SILVA. As in the previous analysis, pairwise comparisons among pipelines revealed widespread differences in the relative abundance of shared genera (*p* < 0.031) (Supplementary Figure 3).

## Discussion

In this paper, we compared four commonly used pipelines, QIIME2, Bioconductor, UPARSE and mothur, run on both Linux and Mac OS, to evaluate how much different bioinformatics pipelines and OS can influence taxonomic classification. In terms of usability of each pipeline (Table 2), we found those implemented in QIIME2 and UPARSE to be the most user-friendly considering that they include only a limited number of commands characterized by simple syntaxes and minimal programming knowledge is required. In contrast, Bioconductor uses the R language, providing an advantageous interface for trained users who are typically familiar with R. Nevertheless, the extensive and detailed documentation of Bioconductor and mothur facilitates their application for users with intermediate command line experience. In the case of UPARSE, this was true except for the taxonomic assignment step, as the UPARSE pipeline does not provide taxonomic assignments to the OTU representative sequences or recommendations on how to run it. The main differences between pipelines consist in the installation procedures. Indeed, USEARCH and mothur only require downloading an executable file, while QIIME2 and Bioconductor require configuration and installation. In particular, as it has been previously noted in a similar study using QIIME1 (D’Argenio et al., 2014), QIIME2 depends on several programs, making the installation much more time consuming and laborious.

**TABLE 2:** Usability of pipelines: pros and cons from an untrained user’s point of view.

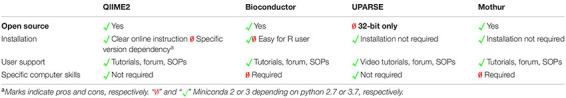

*^*a*^Marks indicate pros and cons, respectively. “
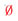
” and “
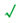
” Miniconda 2 or 3 depending on python 2.7 or 3.7, respectively.*

We have shown that the resulting outputs differed significantly between pipelines despite using the same inputs. In particular, when the clustering was used (i.e., 97% OTU clustering using UPARSE), this resulted in fewer distinct genera in total. This can be due either to the clustering step erroneously clustering distinct genera together due to sequence similarity (which would result in only a single genus being assigned to the OTU), i.e., clustering underestimating the correct number of distinct genera, or due to ASVs sometimes being mis-assigned and resulting in false positives in the list of identified genera. We also found that, despite neither methods employing clustering, processing with Bioconductor led to significantly more detected genera than QIIME2. Examples such as these highlight the fact that measures of bacterial community richness (e.g., alpha- and beta-diversity) cannot be reliably compared for samples processed using different pipelines. For example, the fact that different pipelines result in different numbers of distinct organisms will result in different alpha-diversity values for the same sample. Conducting these comparisons simply using the default pipeline values result in clear differences between pipelines that are difficult to interpret, both in indices of alpha- and beta-diversity (Supplementary Figure 4).

We also found that more reads are unassigned by SILVA when using UPARSE and mothur (which produce OTUs) than QIIME2 (which produces ASVs). OTU clustering generally results in a representative sequence (i.e., cluster centroid) that is taxonomically annotated. Due to the ranking of individual unique 16S rRNA sequences by abundance as the first step of the clustering procedure in UPARSE, this is believed to result in more biologically meaningful representative sequences given the cluster centroid is always the most abundant sequence in the cluster. In contrast, ASVs may allow the detection of potentially important and distinct organisms that may have low prevalence across samples. Theoretically, this may be at the expense of the ability to annotate them taxonomically, but our data do not indicate this. However, since the other ASV-producing method used (Bioconductor) discards unassigned reads prior to the analysis, it is unclear to what extent this difference is due to assignment of ASVs and OTUs, or due to differences in the pipeline.

It was surprising to us that application of the same UPARSE and mothur commands (cf. Supplementary Methods 3, 4) on two different OS (Linux and Mac) produced different results, and we were unable to establish the reason for this observed difference.

Of particular note is the fact that the starkest differences in relative abundances between pipelines were observed in low abundance organisms. For example, *Lentisphaerae* were only detected using QIIME2 and Bioconductor (with relative abundances below 1% in both cases). The problem is further compounded by the fact that low abundance organisms are likely to be less represented in reference databases. Similar observations can be garnered from a previous analysis comparing the MG-RAST and QIIME1 pipelines, where low abundance organisms can differ in their estimated abundance by several orders of magnitude (D’Argenio et al., 2014). This is significant since it is likely that these organisms play nonetheless important roles in human diseases.

It is worth noting that there were clearly discernible phylum-specific biases in certain pipelines: in particular, we observed statistically significant differences between QIIME2 and all other pipelines in *Firmicutes* phylum annotations, and between QIIME2 and all other pipelines except Bioconductor for the *Bacteroidetes* phylum. Thus, different processing pipelines can lead to differences even at the level of phyla, including the two most abundant and prevalent phyla of the human gut microbiota.

A difficulty we identified in our analysis is that certain genera have better inter-pipeline agreement than others. For example, the abundance of the *Faecalibacterium* genus is in good general agreement across outputs from all pipelines, while *Bacteroides* is less so (Figure 5). While we cannot establish the reason behind these differences, we can suggest that they could be likely related to the diversity within these genera: *Faecalibacterium* consists of few species with fewer close relatives, compared to *Bacteroides* which has a great species-level diversity in the human gut microbiota and is therefore more sensitive to bioinformatic steps before taxonomic assignment. Moreover, it is possible that the choice of taxonomic classification method (which are built within different databases) can impact the results differently in different regions of the phylogenetic tree.

Overall, we report differences associated with processing of the same raw data using different bioinformatics pipelines. The magnitude of such differences is comparable to what reported by previous studies investigating the impact of upstream sample processing. Different DNA extraction methods and laboratory locations have been shown to lead to up 10- and 2-fold differences respectively in the relative abundances of specific bacterial genera, respectively (Kennedy et al., 2014). For example, differences in relative abundance of the highly abundant *Bacteroides* genus can be over twofold depending on the extraction method used, and we observed similar differences in magnitude from the sample between different pipelines or even the same pipeline on different OS (Figure 5). Similarly, a comparison between Illumina versus 454 Titanium sequencing platforms resulted in up to twofold difference in the number of detected bacterial genera (Claesson et al., 2010), which is comparable to the difference between Bioconductor and UPARSE or mothur (Figure 4). In addition, a study investigating distinct sample collection and storage methods reported similar (albeit relatively small) differences in the relative abundance of the dominant bacterial phyla (Choo et al., 2015).

## Conclusion

Beyond differences in runtime, the ease of use and installation, we have demonstrated that the use of different bioinformatic pipelines has a strong impact on resulting analyses, with a magnitude that is comparable to differences in upstream sample treatment and sequencing procedures. These differences include the presence of a different number of assigned reads, different number of distinct taxonomies detected in the dataset, and the relative abundance of different organism in the gut microbial community. Taken together, our data indicate that results cannot be compared if obtained by applying different pipelines and that a harmonization is urgent to move forward in the field. As previously noted in the literature, adherence to specific harmonization guidelines would greatly mitigate these difficulties (Nekrutenko and Taylor, 2012). Such guidelines should report the exact versions of software used in an analysis, indicating specific parameters used (even if these are default settings), and standardizing methods and parameter sets within and between research groups. In addition, the field would likely benefit from working as much as possible with open-source, collaborative pipelines and frameworks such as QIIME2, which integrates and is continuously updated with state-of-the-art methods developed in the field. These frameworks can then be automatically applied to the existing set of raw data deposited in public repositories. This may require a shift away from emphasizing static results in an immutable publication format, and toward constantly updated and publicly available databases containing data in both raw and processed forms, such as MG-RAST.

Moreover, particular caution is warranted when conducting meta-analyses combining the results of several studies.

## Data Availability Statement

The raw data supporting the conclusions of this article are publicly available at www.ebi.ac.uk/ena/data/view/PRJEB35434.

## Ethics Statement

The studies involving human participants were reviewed and approved by “Comitato Etico dell’IRCCS San Giovanni di Dio – Fatebenefratelli” (Brescia, Italy) under registration number 57/2014. The patients/participants provided their written informed consent to participate in this study.

## Author Contributions

MMar conceptualized the study and analyzed the data. MMar and TG interpreted the data and contributed equally in writing the manuscript. SP, NL, FR, CF, MMaz, and EM acquired and processed the sample. GG, MS, PM, MF, AS, GF, and AC reviewed and edited the manuscript. GF and AC acquired the funding and guided data interpretation. All authors read and approved the final version of the manuscript.

## Conflict of Interest

The authors declare that the research was conducted in the absence of any commercial or financial relationships that could be construed as a potential conflict of interest.

## Footnotes

1 https://youtu.be/pOV_tuZqzso

2 https://www.drive5.com/usearch/manual/uparse_otu_radius.html

3 www.arb-silva.de/

4 https://zenodo.org/record/1172783#.XtkSOi-ua-y

5 https://zenodo.org/record/801828#.Xh2Uqy3h3AI

6 http://jvenn.toulouse.inra.fr/app/example.html
