# Supplementary material for: Comparison of Bioinformatics Pipelines and Operating Systems for the Analyses of 16S rRNA Gene Amplicon Sequences in Human Fecal Samples
Source: Front Microbiol. 2020 Jun 17;11:1262. doi: 10.3389/fmicb.2020.01262 (PMC7318847; doi:10.3389/fmicb.2020.01262)

Supplementary Material

Supplementary Methods 1. QIIME2 command line. The same version (2018.8) and the same commands were used on Linux and Mac OS. (.txt)

Supplementary Methods 2. Bioconductor command line. The same version (https://f1000research.com/articles/5-1492/v2, 29 OCT 2018) and the same commands were used on Linux and Mac OS. (.txt)

Supplementary Methods 3. UPARSE command line. The same version (11.0.667) and the same commands were used on Linux and Mac OS. (.txt)

Supplementary Methods 4. Mothur command line. The same version (1.43.0) and the same commands were used on Linux and Mac OS. (.txt)

Supplementary Methods 5. Command line used to customize the SILVA (version 132) and RDP (version 16) reference database (version 132) in QIIME2 (https://docs.qiime2.org/2018.8/tutorials/feature-classifier/). The same version (2018.8) and the same commands were used on Linux and Mac OS. (.txt)

Supplementary Methods 6. Command line used to customize the SILVA (version 132) (https://blog.mothur.org/2016/07/07/Customization-for-your-region/) and RDP (version 16) (https://mothur.org/blog/2017/RDP-v16-reference_files/) reference databases in mothur. The same version (1.43.0) and the same commands were used on Linux and Mac OS. (.txt)

Supplementary Table 1. Processed databases. Taxonomic assignment and number of reads as processed by using QIIME2, Bioconductor, UPARSE, or mothur. (.xlsx)

Supplementary Figure 1. Comparison of the relative abundance of phyla obtained by using QIIME2, Bioconductor, UPARSE, or mothur after clustering OTUs at 99%. P values were calculated using Friedman test followed by Dunn's multiple comparisons test. Wilcoxon signed rank test was applied when only 2 pipelines were compared.

Supplementary Figure 2. Comparison of the relative abundance of genera obtained by using QIIME2, Bioconductor, UPARSE, or mothur after clustering OTUs at 99%. P values were calculated using Friedman test followed by Dunn's multiple comparisons test. Wilcoxon signed rank test was applied when only 2 pipelines were compared.

Supplementary Figure 3. Comparison of the relative abundance of genera obtained by using QIIME2, Bioconductor, UPARSE, or mothur after clustering OTUs at 97% and by applying the RDP database (version 16). P values were calculated using Friedman test followed by Dunn's multiple comparisons test. Wilcoxon signed rank test was applied when only 2 pipelines were compared.

Supplementary Figure 4. Alpha (A) and beta diversity (B-C) measures in QIIME2, Bioconductor, UPARSE or mothur. Beta diversity metrics were computed using normalized data except for UPARSE, where they have not been calculated as the free 32-bit version of USEARCH did not allocate enough memory for the analysis. Only one mothur workflow is shown for beta diversity.
P values for Shannon index analysis were calculated using Friedman test followed by Dunn's multiple comparisons test.

Supplementary Methods 1. QIIME2 command line. The same version (2018.8) and the same commands were used on Linux and Mac OS.

```
# Processing performed on cognitively intact persons (here indicated as Healthy Controls, HC) sequencing run
qiime tools import --type SampleData[PairedEndSequencesWithQuality] --input-path manifest_HC.csv --output-path paired-end-demux_HC.qza --input-format PairedEndFastqManifestPhred33
qiime demux summarize --i-data paired-end-demux_HC.qza --o-visualization paired-end-demux_HC.qzv
qiime tools view paired-end-demux_HC.qzv
qiime dada2 denoise-paired --i-demultiplexed-seqs paired-end-demux_HC.qza --p-trim-left-f 17 --p-trunc-len-f 290 --p-trim-left-r 21 --p-trunc-len-r 220 --p-n-threads 0 --output-dir dada2_HC --o-representative-sequences rep-seqs-dada2_HC.qza --o-table table-dada2_HC.qza

# Processing performed on beta amyloid positive AD patients (ABpos) sequencing run
qiime tools import --type SampleData[PairedEndSequencesWithQuality] --input-path manifest_ABpos.csv --output-path paired-end-demux_ABpos.qza --input-format PairedEndFastqManifestPhred33
qiime demux summarize --i-data paired-end-demux_ABpos.qza --o-visualization paired-end-demux_ABpos.qzv
qiime tools view paired-end-demux_ABpos.qzv
qiime dada2 denoise-paired --i-demultiplexed-seqs paired-end-demux_ABpos.qza --p-trim-left-f 17 --p-trunc-len-f 290 --p-trim-left-r 21 --p-trunc-len-r 220 --p-n-threads 0 --output-dir dada2_ABpos --o-representative-sequences rep-seqs-dada2_ABpos.qza --o-table table-dada2_ABpos.qza

# Merging tables and sequences
qiime feature-table merge --i-tables table-dada2_HC.qza --i-tables table-dada2_ABpos.qza --o-merged-table table-dada2_all.qza
qiime feature-table merge-seqs --i-data rep-seqs-dada2_HC.qza --i-data rep-seqs-dada2_ABpos.qza --o-merged-data rep-seqs-dada2_all.qza
qiime feature-table summarize --i-table table-dada2_all.qza --o-visualization table-dada2_all.qzv --m-sample-metadata-file sample-metadata.tsv
qiime feature-table tabulate-seqs --i-data rep-seqs-dada2_all.qza --o-visualization rep-seqs-dada2_all.qzv
qiime feature-classifier classify-sklearn --i-classifier /silva-V3V4-classifier.qza --i-reads rep-seqs-dada2_all.qza --o-classification taxonomy.qza
qiime metadata tabulate --m-input-file taxonomy.qza --o-visualization taxonomy.qzv
qiime taxa barplot --i-table table-dada2_all.qza --i-taxonomy taxonomy.qza --m-metadata-file sample-metadata.tsv --o-visualization taxa-bar-plots.qzv
```

Supplementary Methods 2. Bioconductor command line. The same version (https://f1000research.com/articles/5-1492/v2, 29 OCT 2018) and the same commands were used on Linux and Mac OS.

```
set.seed(100)
# Processing performed on HC sequencing run
fns_HC <- sort(list.files(miseq_path, full.names = TRUE))
fnFs_HC <- fns_HC[grepl("R1", fns_HC)]
```

```
fnRs_HC <- fns_HC[grepl("R2", fns_HC)]
plotQualityProfile(fnFs_HC[1:3])
plotQualityProfile(fnRs_HC[1:3])
if(!file_test("-d", filt_path)) dir.create(filt_path)
filtFs_HC <- file.path(filt_path, basename(fnFs_HC))
filtRs_HC <- file.path(filt_path, basename(fnRs_HC))
for(i in seq_along(fnFs_HC)) {
  fastqPairedFilter(c(fnFs_HC[[i]], fnRs_HC[[i]]),
                    c(filtFs_HC[[i]], filtRs_HC[[i]]),
                truncLen=c(290,220), trimLeft=c(17,21), maxN=0, maxEE=c(2,5),
truncQ=2,compress=TRUE, verbose=TRUE)
}
derepFs_HC <- derepFastq(filtFs_HC, verbose=TRUE)
derepRs_HC <- derepFastq(filtRs_HC, verbose=TRUE)
errF_HC <- learnErrors(filtFs_HC, multithread=TRUE)
errR_HC <- learnErrors(filtRs_HC, multithread=TRUE)
plotErrors(errF_HC)
plotErrors(errR_HC)
dadaFs_HC<-dada(derepFs_HC, err=errF_HC, pool=TRUE, multithread=TRUE)
dadaRs_HC<-dada(derepRs_HC, err=errR_HC, pool=TRUE, multithread=TRUE)
mergers_HC<-mergePairs(dadaFs_HC, derepFs_HC, dadaRs_HC, derepRs_HC, verbose=TRUE)
seqtab_HC<- makeSequenceTable(mergers_HC, orderBy="abundance")

# Processing performed on ABpos sequencing run
fns_ABpos <- sort(list.files(miseq_path, full.names = TRUE))
fnFs_ABpos <- fns_ABpos[grepl("R1", fns_ABpos)]
fnRs_ABpos <- fns_ABpos[grepl("R2", fns_ABpos)]
plotQualityProfile(fnFs_ABpos[1:3])
plotQualityProfile(fnRs_ABpos[1:3])
if(!file_test("-d", filt_path)) dir.create(filt_path)
filtFs_ABpos <- file.path(filt_path, basename(fnFs_ABpos))
filtRs_ABpos <- file.path(filt_path, basename(fnRs_ABpos))
for(i in seq_along(fnFs_ABpos)) {
  fastqPairedFilter(c(fnFs_ABpos[[i]], fnRs_ABpos[[i]]),
                    c(filtFs_ABpos[[i]], filtRs_ABpos[[i]]),
                truncLen=c(290,220), trimLeft=c(17,21), maxN=0, maxEE=c(2,5),
truncQ=2,compress=TRUE, verbose=TRUE)
}
derepFs_ABpos <- derepFastq(filtFs_ABpos, verbose=TRUE)
derepRs_ABpos <- derepFastq(filtRs_ABpos, verbose=TRUE)
errF_ABpos <- learnErrors(filtFs_ABpos, multithread=TRUE)
errR_ABpos <- learnErrors(filtRs_ABpos, multithread=TRUE)
plotErrors(errF_ABpos)
plotErrors(errR_ABpos)
dadaFs_ABpos<-dada(derepFs_ABpos, err=errF_ABpos, pool=TRUE, multithread=TRUE)
dadaRs_ABpos<-dada(derepRs_ABpos, err=errR_ABpos, pool=TRUE, multithread=TRUE)
mergers_ABpos<-mergePairs(dadaFs_ABpos, derepFs_ABpos, dadaRs_ABpos, derepRs_ABpos,
verbose=TRUE)
```

```
seqtab_ABpos<- makeSequenceTable(mergers_ABpos, orderBy="abundance")

# Merging results
seqtab.all<- mergeSequenceTables(seqtab_HC, seqtab_ABpos)
seqtabNoC<- removeBimeraDenovo(seqtab.all, verbose=TRUE)
ref_fasta<-"/silva_nr_v132_train_set.fa.gz"
taxtab<- assignTaxonomy(seqtabNoC, refFasta = ref_fasta, multithread=TRUE, verbose=TRUE)
colnames(taxtab)<-c("Kingdom","Phylum","Class","Order","Family","Genus")
write.table(taxtab, "seqtabNoC_w_taxonomy.txt", sep='\t', row.names=FALSE, quote=FALSE)
seqs <- getSequences(seqtabNoC)
names(seqs) <- seqs
alignment <- AlignSeqs(DNAStringSet(seqs), anchor=NA)
phang.align <- phyDat(as(alignment, "matrix"), type="DNA")
dm <- dist.ml(phang.align)
treeNJ <- NJ(dm) # Note, tip order != sequence order
fit = pml(treeNJ, data=phang.align)
fitGTR <- update(fit, k=4, inv=0.2)
fitGTR <- optim.pml(fitGTR, model="GTR", optInv=TRUE, optGamma=TRUE,
        rearrangement = "stochastic", control = pml.control(trace = 0))
samdf <- read.csv("metadata.csv",header=TRUE)
samdf <- samdf[!duplicated(samdf$SampleID),]
all(rownames(seqtabNoC) %in% samdf$SampleID)
rownames(samdf) <- samdf$SampleID
ps <- phyloseq(tax_table(taxtab), sample_data(samdf), otu_table(seqtabNoC, taxa_are_rows =
FALSE),phy_tree(fitGTR$tree))
```

Supplementary Methods 3. UPARSE and UNOISE command line. The same version (11.0.667) and the same commands were used on Linux and Mac OS.

```
usearch -fastq_mergepairs *R1*.fastq -relabel @ -fastq_minmergelen 400 -fastq_maxmergelen 480 -
fastqout ../output/merged.fq -fastq_maxdiffs 10
usearch -fastx_truncate merged.fq -stripleft 17 -stripright 21 -fastqout stripped.fq
usearch -fastq_filter stripped.fq -fastq_maxee 1.0 -fastaout filtered.fa
usearch -fastx_uniques filtered.fa -fastaout uniques.fa -relabel Uniq -sizeout
#Clustering step using UPARSE
usearch -cluster_otus uniques.fa -minsize 2 -otus otu.fa -relabel Otu
usearch -usearch_global merged.fq -db otu.fa -strand plus -id 0.97 -otutabout otutable.txt
#Taxonomy assignation using mothur
classify.seqs(fasta=otu.fasta, count=otu.count_table, template= /silva.v34.fasta, taxonomy=/
silva.seed_v132.tax, cutoff=80)
remove.lineage(fasta=otu.fasta, count=otu.count_table, taxonomy=otu.seed_v132.wang.taxonomy,
taxon=unknown;-Archaea;-Eukaryota;)
summary.tax(taxonomy=otu.seed_v132.wang.pick.taxonomy, count=otu.pick.count_table)
phylotype(taxonomy=otu.seed_v132.wang.pick.taxonomy)
classify.otu(list=otu.seed_v132.wang.pick.tx.list, count=otu.pick.count_table,
taxonomy=otu.seed_v132.wang.pick.taxonomy, label=0.03, cutoff=80, probs=F)
#Clustering step using UNOISE
usearch -unoise3 uniques.fa -zotus otus100.fa -tabbedout unoise3.txt
```

usearch -cluster_smallmem otus100.fa -id 0.99 -centroids otus99.fa -sortedby other
#Taxonomy assignation using mothur
classify.seqs(fasta=otus99.fa, count=otus99.count_table, template= /silva.v34.fasta, taxonomy=/silva.seed_v132.tax, cutoff=80)
remove.lineage(fasta=otus99.fa, count=otus99.count_table, taxonomy=otus99.seed_v132.wang.taxonomy, taxon=unknown;-Archaea;-Eukaryota;)
summary.tax(taxonomy=otus99.seed_v132.wang.pick.taxonomy, count=otus99.pick.count_table)
phylotype(taxonomy=otu.seed_v132.wang.pick.taxonomy)
classify.otu(list=otus99.seed_v132.wang.pick.tx.list, count=otus99.pick.count_table, taxonomy=otus99.seed_v132.wang.pick.taxonomy, label=0.01, cutoff=80, probs=F)

Supplementary Methods 4. Mothur command line. The same version (1.43.0) and the same commands were used on Linux and Mac OS.
make.file(inputdir='/input/', type=fastq, prefix=stability)
make.contigs(file=stability.files)
summary.seqs(fasta=stability.trim.contigs.fasta)
trim.seqs(fasta=stability.trim.contigs.fasta, oligos=v34.oligos)
screen.seqs(fasta=stability.trim.contigs.trim.fasta, group=stability.contigs.groups, maxambig=0)
summary.seqs(fasta=stability.trim.contigs.trim.good.fasta)
unique.seqs(fasta=stability.trim.contigs.trim.good.fasta)
count.seqs(name=stability.trim.contigs.trim.good.names, group=stability.contigs.good.groups)
summary.seqs(count=stability.trim.contigs.trim.good.count_table)
align.seqs(fasta=stability.trim.contigs.trim.good.unique.fasta, reference=/silva.v34.fasta, flip=T)
summary.seqs(fasta=stability.trim.contigs.trim.good.unique.align, count=stability.trim.contigs.trim.good.count_table)
screen.seqs(fasta=stability.trim.contigs.trim.good.unique.align, count=stability.trim.contigs.trim.good.count_table, summary=stability.trim.contigs.trim.good.unique.summary, start=40, end=17052, maxhomop=8)
filter.seqs(fasta=stability.trim.contigs.trim.good.unique.good.align, vertical=T, trump=.)
unique.seqs(fasta=stability.trim.contigs.trim.good.unique.good.filter.fasta, count=stability.trim.contigs.trim.good.good.count_table)
pre.cluster(fasta=stability.trim.contigs.trim.good.unique.good.filter.unique.fasta, count=stability.trim.contigs.trim.good.unique.good.filter.count_table, diffs=4)
chimera.vsearch(fasta=stability.trim.contigs.trim.good.unique.good.filter.unique.precluster.fasta, count=stability.trim.contigs.trim.good.unique.good.filter.unique.precluster.count_table, dereplicate=t, processors=8)
remove.seqs(fasta=stability.trim.contigs.trim.good.unique.good.filter.unique.precluster.fasta, accnos=stability.trim.contigs.trim.good.unique.good.filter.unique.precluster.denovo.vsearch.accnos)
classify.seqs(fasta=stability.trim.contigs.trim.good.unique.good.filter.unique.precluster.pick.fasta, count=stability.trim.contigs.trim.good.unique.good.filter.unique.precluster.denovo.vsearch.pick.count_table, reference=silva.v34.fasta, taxonomy=silva.seed_v132.tax, cutoff=80)
remove.lineage(fasta=stability.trim.contigs.trim.good.unique.good.filter.unique.precluster.pick.fasta, count=stability.trim.contigs.trim.good.unique.good.filter.unique.precluster.denovo.vsearch.pick.count_table, taxonomy=stability.trim.contigs.trim.good.unique.good.filter.unique.precluster.pick.seed_v132.wang.taxonomy, taxon=taxon=unknown;-Archaea;-Eukaryota;)
summary.tax(taxonomy=current, count=current)
#Clustering step: 97% identity threshold

```
cluster.split(fasta=stability.trim.contigs.trim.good.unique.good.filter.unique.precluster.pick.pick.fasta
,
count=stability.trim.contigs.trim.good.unique.good.filter.unique.precluster.denovo.vsearch.pick.pick.
count_table,
taxonomy=stability.trim.contigs.trim.good.unique.good.filter.unique.precluster.pick.seed_v132.wang
.pick.taxonomy, splitmethod=classify, taxlevel=4, cutoff=0.03)
make.shared(list=stability.trim.contigs.trim.good.unique.good.filter.unique.precluster.pick.pick.opti_
mcc.list,
count=stability.trim.contigs.trim.good.unique.good.filter.unique.precluster.denovo.vsearch.pick.pick.
count_table, label=0.03)
classify.otu(list=stability.trim.contigs.trim.good.unique.good.filter.unique.precluster.pick.pick.opti_
mcc.list,
count=stability.trim.contigs.trim.good.unique.good.filter.unique.precluster.denovo.vsearch.pick.pick.
count_table,
taxonomy=stability.trim.contigs.trim.good.unique.good.filter.unique.precluster.pick.seed_v132.wang
.pick.taxonomy, label=0.03, cutoff=80, probs=F)
phylotype(taxonomy=stability.trim.contigs.trim.good.unique.good.filter.unique.precluster.pick.seed_
v132.wang.pick.taxonomy)
#Clustering step: 99% identity threshold
cluster.split(fasta=stability.trim.contigs.trim.good.unique.good.filter.unique.precluster.pick.pick.fasta
,
count=stability.trim.contigs.trim.good.unique.good.filter.unique.precluster.denovo.vsearch.pick.pick.
count_table,
taxonomy=stability.trim.contigs.trim.good.unique.good.filter.unique.precluster.pick.seed_v132.wang
.pick.taxonomy, splitmethod=classify, taxlevel=4, cutoff=0.01)
make.shared(list=stability.trim.contigs.trim.good.unique.good.filter.unique.precluster.pick.pick.opti_
mcc.list,
count=stability.trim.contigs.trim.good.unique.good.filter.unique.precluster.denovo.vsearch.pick.pick.
count_table, label=0.01)
classify.otu(list=stability.trim.contigs.trim.good.unique.good.filter.unique.precluster.pick.pick.opti_
mcc.list,
count=stability.trim.contigs.trim.good.unique.good.filter.unique.precluster.denovo.vsearch.pick.pick.
count_table,
taxonomy=stability.trim.contigs.trim.good.unique.good.filter.unique.precluster.pick.seed_v132.wang
.pick.taxonomy, label=0.01, cutoff=80, probs=F)
```

Supplementary Methods 5. Command line used to customize the SILVA (version 132) and RDP (version 16) reference databases in QIIME2 (https://docs.qiime2.org/2018.8/tutorials/feature-classifier/). The same version (2018.8) and the same commands were used on Linux and Mac OS.

```
#SILVA
qiime feature-classifier extract-reads --i-sequences 99-otus.qza --p-f-primer
CCTACGGGNGGCWGCAG --p-r-primer GGATTAGATACCCVHGTAGTC --o-reads ref-
seqs.qza
qiime feature-classifier fit-classifier-naive-bayes --i-reference-reads ref-seqs.qza --i-reference-
taxonomy 7_level_taxonomy.qza --o-classifier silva-V3V4-classifier.qza
#RDP
```

#RDP database download and conversion for QIIME2 feature-classifier as in https://vdreis.com/rdp-database/
qiime feature-classifier extract-reads --i-sequences RDP.qza --p-f-primer CCTACGGGNGGCWGCAG --p-r-primer GGATTAGATACCCVHGTAGTC --o-reads ref-seqs.qza
qiime feature-classifier fit-classifier-naive-bayes --i-reference-reads ref-seqs.qza --i-reference-taxonomy ref-RDP_tax_new.qza --o-classifier RDP16-V3V4-classifier.qza

Supplementary Methods 6. Command line used to customize the SILVA (version 132) (https://blog.mothur.org/2016/07/07/Customization-for-your-region/) and RDP (version 16) reference databases (https://mothur.org/blog/2017/RDP-v16-reference_files/) in mothur. The same version (1.43.0) and the same commands were used on Linux and Mac OS.
#SILVA
wget -N https://www.arb-silva.de/fileadmin/arb_web_db/release_132/ARB_files/SILVA_132_SSURef_NR99_13_12_17_opt.arb.gz
align.seqs(fasta=ecoli_v3v4.fasta, reference=silva.seed_v132.align)
summary.seqs(fasta=ecoli_v3v4.align)
pcr.seqs(fasta= /silva.seed_v132.align, start=6388, end=25316, keepdots=FALSE)
rename.file(input=silva.seed_v132.pcr.align, new=silva.v34.fasta)
summary.seqs(fasta=silva.v34.fasta)

Supplementary Figure 1. Comparison of the relative abundance of phyla obtained by using QIIME2, Bioconductor, UPARSE, or mothur after clustering OTUs at 99%.
P values were calculated using Friedman test followed by Dunn's multiple comparisons test. Wilcoxon signed rank test was applied when only 2 pipelines were compared.

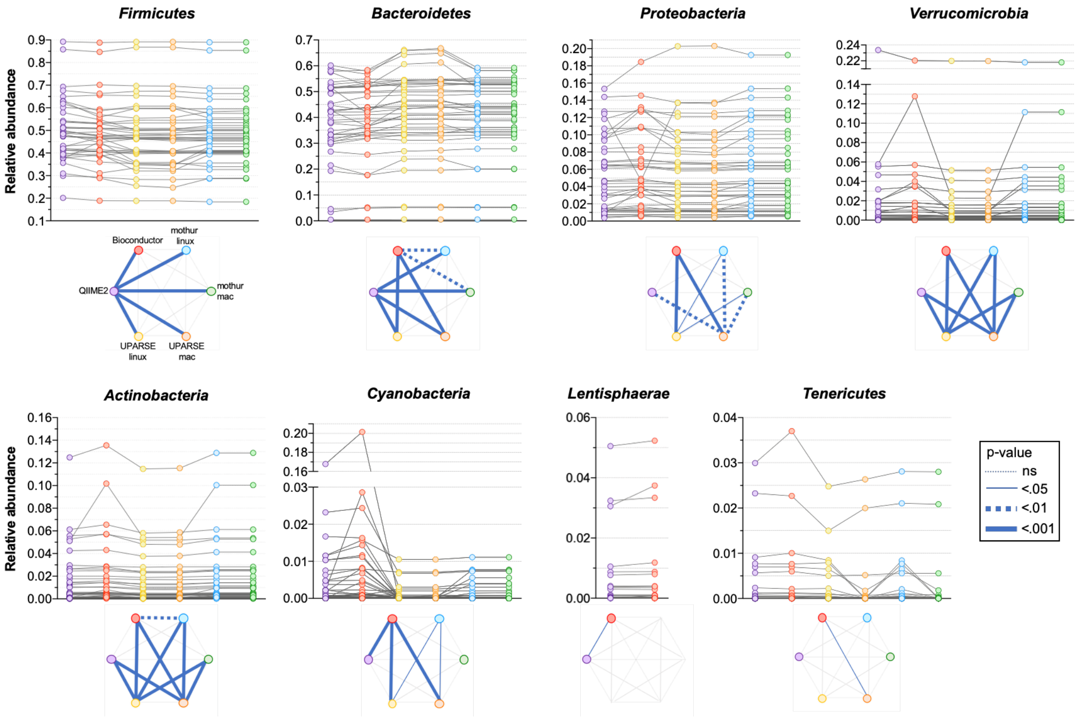

Supplementary Figure 2. Comparison of the relative abundance of genera obtained by using QIIME2, Bioconductor, UPARSE, or mothur after clustering OTUs at 99%.
P values were calculated using Friedman test followed by Dunn's multiple comparisons test. Wilcoxon signed rank test was applied when only 2 pipelines were compared.

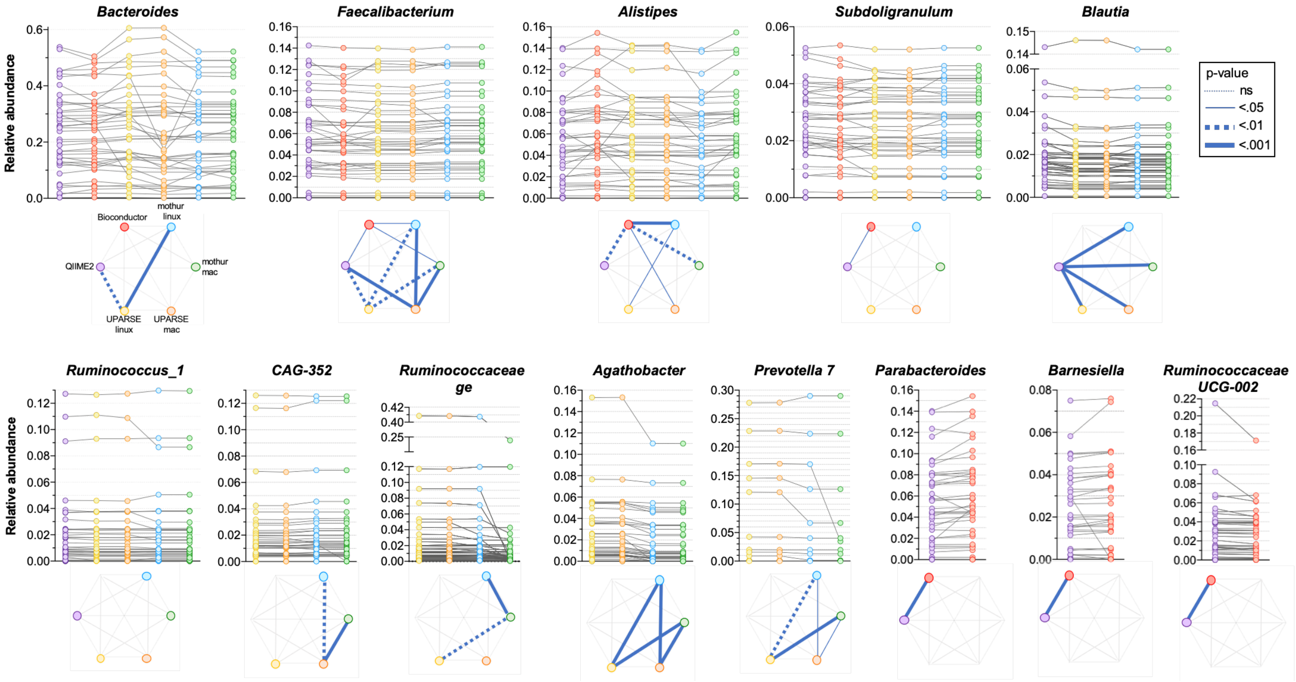

Supplementary Figure 3. Comparison of the relative abundance of genera obtained by using QIIME2, Bioconductor, UPARSE, or mothur after clustering OTUs at 97% and by applying the RDP database (version 16).

P values were calculated using Friedman test followed by Dunn's multiple comparisons test. Wilcoxon signed rank test was applied when only 2 pipelines were compared.

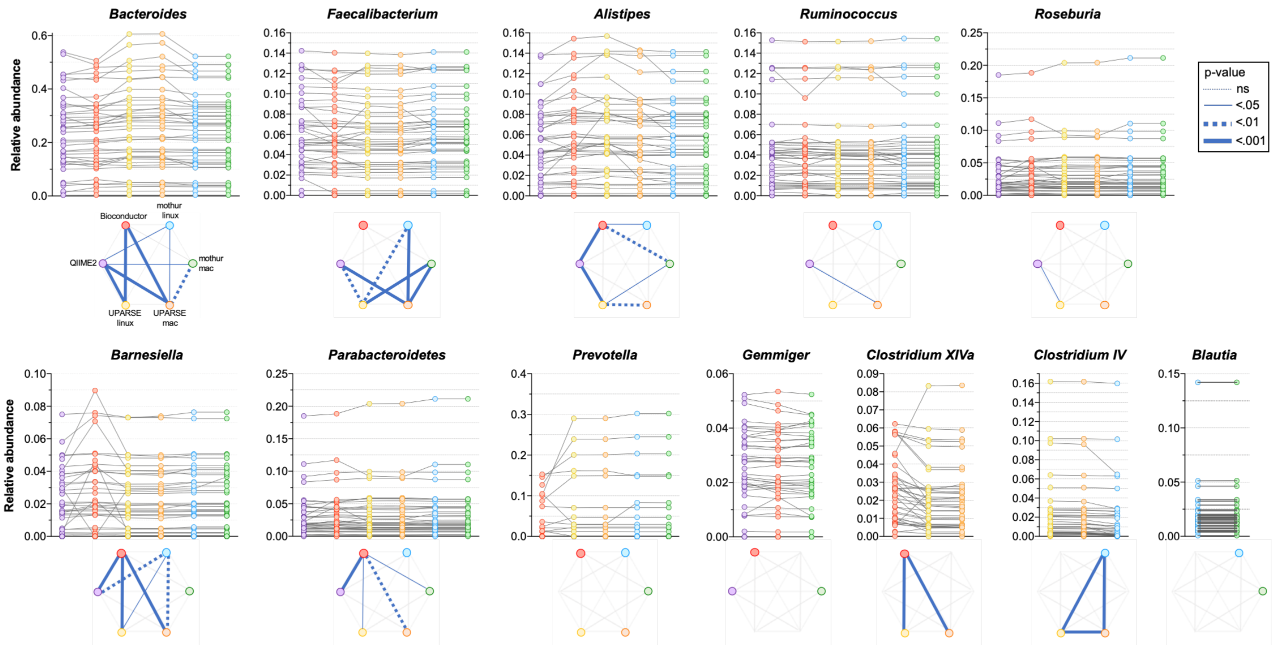

Supplementary Figure 4. Alpha (A) and beta diversity (B-C) measures in QIIME2, Bioconductor, UPARSE or mothur. Beta diversity metrics were computed using normalized data except for UPARSE, where they have not been calculated as the free 32-bit version of USEARCH did not allocate enough memory for the analysis. Only one mothur workflow is shown for beta diversity.
P values for Shannon index analysis were calculated using Friedman test followed by Dunn's multiple comparisons test.

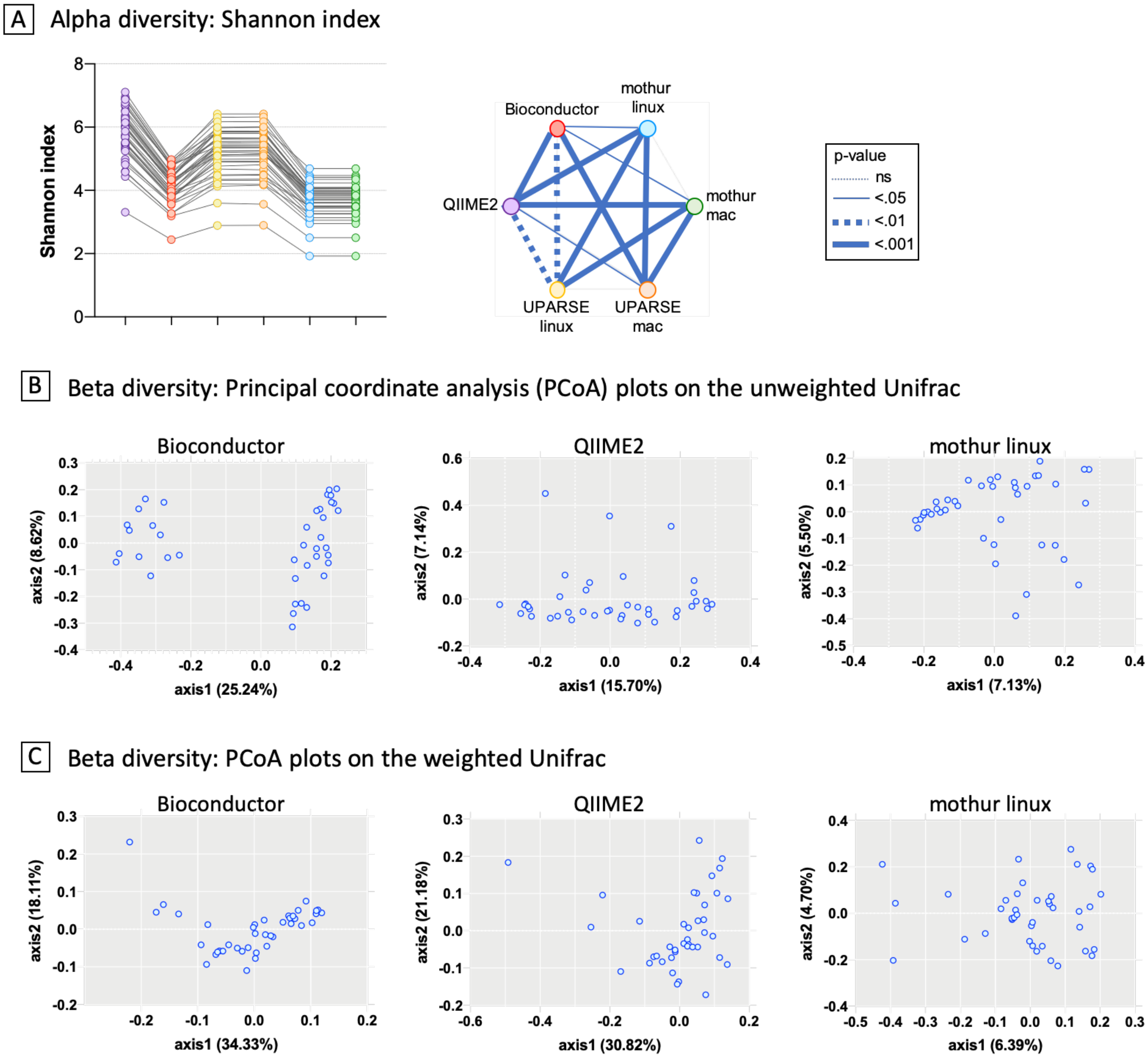

Supplement: Supplementary file 1 [file Data_Sheet_1.pdf]
